# In Silico functional and phylogenetic analyses of fungal immunomodulatory proteins of some edible mushrooms

**DOI:** 10.1186/s13568-022-01503-w

**Published:** 2022-12-26

**Authors:** Ayyagari Ramlal, Aveek Samanta

**Affiliations:** 1 Department of Botany, University of Delhi, New Delhi, Delhi 110007 India; 2 School of Biological Sciences, Universiti Sains Malaysia (USM), 11800 Georgetown, Penang Malaysia; 3 Department of Botany, Prabhat Kumar College, Contai, 721401 West Bengal India

**Keywords:** Mushrooms, Traditional medicines, Fungal immunomodulatory proteins (FIPs), Motifs, Bioactive peptides, Diseases

## Abstract

Mushrooms are a well known source of many bioactive and nutritional compounds with immense applicability in both the pharmaceutical and food industries. They are widely used to cure various kinds of ailments in traditional medicines. They have a low amount of fats and cholesterol and possess a high number of proteins. Immunomodulators have the ability which can improve immunity and act as defensive agents against pathogens. One such class of immunomodulators is fungal immunomodulatory proteins (FIPs). FIPs have potential roles in the treatment of cancer, and immunostimulatory effects and show anti-tumor activities. In the current study, 19 FIPs from edible mushrooms have been used for comparison and analysis of the conserved motifs. Phylogenetic analysis was also carried out using the FIPs. The conserved motif analysis revealed that some of the motifs strongly supported their identity as FIPs while some are novel. The fungal immunomodulatory proteins are important and have many properties which can be used for treating ailments and diseases and this preliminary study can be used for the identification and functional characterization of the proposed novel motifs and in unraveling the potential roles of FIPs for developing newer drugs.

**Supplementary Information:**

The online version contains supplementary material available at 10.1186/s13568-022-01503-w.

## Introduction

Mushrooms have a plethora of nutraceutical compounds and also contain therapeutic compounds which prevent many diseases (Khatum et al. 2012; Bains et al. 2021). Mushrooms are a prominent source of nutrients which include carbohydrates, proteins (3.5–4% wet weight and 19–35% on dry weight), contain essential amino acids like lysine and valine, and minerals (Ca, Fe, Mn, Zn, Se and Mg) and vitamins such as B complex, C, D and folic acid along with fibers (Hesami et al. 2014; Rahi and Malik 2016; Mbuge and Mutai 2018; Naseem et al. 2020). They are also a source of constituents like glycoproteins, proteoglycans, lectins, quinones, terpenes, alkaloids, steroids, polysaccharides, and lanostanoids (Wang et al. 2012; Bains et al. 2021). They possess excellent pharmacological properties like antioxidant, antitumor, antimicrobial, prevent hypertension, anti-inflammatory, antidiabetic, anticancerous, antiviral, and maintain cholesterol levels (Khatum et al. 2012; Mallikarjuna et al. 2013; Bains et al. 2021). Mushrooms also possess immunomodulatory effects, radical scavenging, and antibacterial and cardioprotective properties (Wang et al. 2012; Bains et al. 2021). Mushrooms are also being used in the Chinese Traditional Medicine (TCM) and antedate history (3000 BC) along with this it is also used as a functional food in Japan and other countries (Wang et al. 2012; Xu et al. 2011). Nowadays, mushrooms produce many biologically active compounds which include ribosome-inactivating proteins (RIPs), lectins, ribonuclease, laccases, proteins having antimicrobial properties, and fungal immunomodulatory proteins (FIPs). Many recent studies have shown that mushrooms have immunomodulatory properties which include polysaccharides, terpenes, proteins, and sterols, etc. ${\upbeta }$1 → 3D-glucans, and their derivatives and different types of proteins are being investigated for their immune-stimulating effects. For instance, these compounds promote mitosis and differentiation of hematopoietic stem cells and activate immune effector cells (human peripheral blood mononuclear cells—hPBMC) (Wang et al. 2012). Fungal immunomodulatory proteins are small proteins (bioactive compounds) of low molecular weight which are isolated from mushrooms as they possess high medicinal values in the treatment of tumours, and cancers and helps to promote or enhance the immune (Valverde et al. 2015).

### Fungal immunomodulatory proteins (FIPs)

The FIPs are a new class of proteins that have immense potential in the formation of newer drugs or compounds for various diseases (Xu et al. 2011; Liu et al. 2020). Mushrooms are a storehouse of many bioactive compounds and macromolecules like polysaccharides, and proteins. Along with the small molecules and some complex molecules polysaccharide-protein complexes, glycoproteins, proteoglycans, terpene compounds, etc. (Wang et al. 2012; Xu et al. 2011). Many mushrooms produce proteins known as immunomodulators that are potential targets for immune cells of humans affecting both innate and adaptive systems and can be used for various therapeutic purposes. These proteins play a major role in the inhibition of cell growth and proliferation, induce autophagy and apoptosis and reduce the aggression of invasion and migration of tumor-causing cells (Li et al. 2018; Chalamaiah et al. 2018). These are known as fungal immunomodulatory proteins (FIPs) or immune-boosting compounds and also immunomodulators. The FIPs are small molecular weight proteins ranging from 12 to 13 kDa having around 110–114 amino acids. Asp and Val are found in abundance more than other amino acids and lack Cys, His, and Met residues (Qu et al. 2018; Ejike et al. 2020). Amino acid, Asp acts as a common site for carbohydrate attachment required for the formation of glycoproteins while Val is essential amino acid having stimulatory activity. FIPs can be glycosylated (LZ-8) or non-glycosylated (FIP-Vvo, FIP-Fve and FIP-Gts) (Ejike et al. 2020). It consists of 1–3 α-helixes, 7–9 β-sheets, and random coils (Liu et al. 2020). The FIPs are involved in the activation and proliferation of macrophages, immune regulation along with maturation of dendritic cells and activation of T cells in mouse (Ejike et al. 2020; Li et al. 2021). As described by Liu et al. (2020), the type of the FIPs observed in the current study are of the Fve-type FIPs, one of the domain-type FIPs due to the presence of signature Pfam identity (PF09259). This is the major type of FIP which is extensively studied which include anti-cancer, anti-tumor, cytokine production, immunomodulatory and hemagglutinating abilities (Li et al. 2011; Liu et al. 2020). The other types of FIPs include Cerato-type FIPs, PCP-like FIPs, TFP-like FIPs, and unclassified FIPs whose details are not much yet understood (Liu et al. 2020).

Some of the known FIPs include FIP-glu or Ling Zhi – 8 (*Ganoderma lucidium*), FIP-fve (*Flammulina velutipes*), FIP-gts (*Ganoderma tsugae*), FIP-vvo (*Volvoriella volvacea*) (Xu et al. 2011). *G. lucidum* (Kino et al. 1989, NCBI:txid5315), *G. tsugae* (Lin et al. 1997, NCBI:txid34467), *G. japoncium* (Lin et al. 2009, NCBI:txid36072), *G. microsporum* (Wu et al. 2007, NCBI:txid34462), *G. applanatum* (Agricultural Culture Collection of China, ACCC: Strain Nu. 51,348), *G. atrum* (Xu et al. 2016, NCBI:txid1188807), *G. sinense* (Zhou et al. 2009, NCBI:txid36075), *G. boninense* (Strain NJ3), *G. amboinense* (Jiang et al. 2016), G. *tenus* (ACCC: Strain Nu. 50602), and *G. capense* (ACCC: Strain Nu. 51229), which are named as LZ-8 (FIP-glu), FIP-gts, FIP-gja, FIP-gmi, FIP-gap1, FIP-gap2, FIP-gas, FIP-gsi, FIP-gbo, FIP-gam, FIP-gte, and FIP-gca, respectively. More than 600 genes are involved in 96 biotic stress-related processes in antitumor activity or action through the FIPs (Li et al. 2019). Immunity is essential for survival and provides protection against invading foreign agents like microorganisms including bacteria, and viruses, or maybe against stress, lifestyle practices, and antigens (Chalamaiah et al. 2018). Since, the outbreak of coronavirus (Khan et al. 2021; Ahmad et al. 2021 reviewed the importance of technology in the detection and prevention of the virus) similarly, immune-boosting and antiviral compounds came in limelight and their usage is promoted and gained the attention due to their potential health-promoting factors (Cai et al. 2020; Khavinson et al. 2020; Pavlicevic et al. 2022). Though many of the sequences are yet unknown and need to be identified for the purpose of drug development and as a functional food.

The study deals with the in-silico analyses of the fungal immunomodulatory proteins (FIPs) or immune-boosting compounds from edible mushrooms. Subsequently, protein and gene features and characteristics and evolutionary divergence were studied.

## Materials and methods

### Sequence analysis of FIPs protein family in edible mushrooms

The amino acid sequences of the fungal immunomodulatory proteins (FIPs) were retrieved using BLAST of NCBI (https://blast.ncbi.nlm.nih.gov/BlastAlign.cgi), UNIPROT (https://www.uniprot.org/) (Ramlal et al. 2021) and as given in Table 1. The FIP sequence of *Ganoderma applanatum* (FIP-Gapp1; Table 1) was used to retrieve the FIP sequences of only edible mushrooms using the NCBI BLAST program with an E < 0.0000000001 ($1{\text{E}}^{-10}$) and > 55% percent identity. Second, the candidate sequences were then confirmed by the Pfam FIP motif (PF09259) using the HMMER software (http://hmmer.org/) and SMART (http://smart.embl-heidelberg.de/) (Letunic et al. 2021) (Additional file 3: Table S1).

**Table 1:** List of 19 FIPs amino acid sequences from different edible mushrooms and their sequence properties

Accession no	Protein	Species	Family	Given name	Length	MW (Da)	PI	Cellular location	Signal Peptide	References
AEP68179.1	Fungal immunomodulatory protein	*Ganoderma applanatum*	Ganodermataceae	Fip-Gapp1	113	12741.2	4.8	E	N	NCBI, Qu et al. 2018
ART88472.1	Fungal immunomodulatory protein FIP-gap2	*Ganoderma applanatum*	Ganodermataceae	Fip-Gapp2	113	12518.2	4.86	C/E	N	NCBI, Qu et al. 2018
AJD79556.1	Immunomodulatory protein	*Ganoderma atrum*	Ganodermataceae	Fip-Gat	111	12450.93	4.8	E	N	NCBI, Qu et al. 2018
AAX98241.1	Immunomodulatory protein	*Ganoderma japonicum*	Ganodermataceae	Fip-Gjo	111	12478.94	4.62	E	N	NCBI, Qu et al. 2018
AAA33350.1	Immunomodulatory protein.	*Ganoderma lucidum*	Ganodermataceae	Fip-Glu	111	12509.95	4.84	E	N	NCBI, Qu et al. 2018
P80412^a^	Immunomodulatory protein FIP-Fve	*Flammulina velutipes*	Physalacriaceae	Fip-Fve	114	12704.28	6.14	E/Cl	N	UniProt, Ko et al. 1995
-	Immunomodulatory protein	*Ganoderma tsugae*	Ganodermataceae	Fip-Gts	110	12378.76	4.84	E	N	Lin et al. 1997
-	Immunomodulatory protein	*Volvariella volvacea*	Pluteaceae	Fip-Vvo	112	12667.23	7.83	E	Y	Hsu et al. 1997
AKU37620.1	Fungal immunomodulatory protein	*Chroogomphis rutilus*	Gomphidiaceae	Fip-Cru	113	12651.13	4.93	E	N	NCBI, Li et al. 2020
E7FH75^a^	Chain A, an immunomodulatory protein	*Ganoderma microsporum*	Ganodermataceae	Fip-Gmi	134	15170.79	5.13	E	N	NCBI, Li et al. 2020
MW280109.1^b^	Immunomodulatory protein	*Ganoderma tenue*	Ganodermataceae	Fip-Gte	111	12522.0	4.84	E	N	NCBI
UOF75531.1	Immunomodulatory protein	*Ganoderma capense*	Ganodermataceae	Fip-Gca	111	12414.84	4.44	E	Y	NCBI
QMV83132.1	Immunomodulatory protein	*G*. sp TQC-2021a	Ganodermataceae	Fip-G	111	12420.9	4.8	E	N	NCBI
KAI1793471.1	Immunomodulatory protein 8	*G leucocontextum*	Ganodermataceae	Fip-Gle	112	12624.18	4.73	E	N	NCBI
AUB29452.1	Partial Immunomodulatory protein	*G. resinaceum*	Ganodermataceae	Fip-Gre	111	12458.95	5.14	E	N	NCBI
TFK85195.1	Immunomodulatory protein FIP-Fve	*Polyporus arcularis* HHB13444	Polyporaceae	Fip-Par	112	12338.75	7.85	E	N	NCBI
RPD64156.1	Immunomodulatory protein FIP-Fve	*Lentinus tigrinus* ALCF2SS1-6	Polyporaceae	Fip-Lti	112	12613.14	8.66	E	Y	NCBI
KAI0695342.1	Immunomodulatory protein FIP-Fve	*Cerioporus squamosus*	Polyporaceae	Fip-Csq	112	12327.66	5.44	E	N	NCBI
KAI0063119.1	Immunomodulatory protein	*Artomyces pyxidatus*	Auriscalpiaceae	Fip-Apy	120	13581.24	7.86	E	N	NCBI

^a^UNIPROT ID, ^b^GenBank ID, C – Cytoplasmic, Cl – Chloroplastic, E – Extracellular, N – No and Y – Yes

### Motif, conserved domain, physiochemical properties, multiple sequence alignment (MSA) phylogenetic and toxicity analyses FIPs

#### Physiochemical properties

The physiochemical data like length, molecular weight, and theoretical isoelectric points (pIs) of FIP sequences were analyzed by the ProtParam server of Expasy (https://web.expasy.org/protparam/). The cellular localization was predicted using WoLF PSORT (https://www.genscript.com/tools/wolf-psort) (Horton et al. 2007), CELLO (http://cello.life.nctu.edu.tw/) (Yu et al. 2006) and TargetP2.0 (https://services.healthtech.dtu.dk/service.php?TargetP-2.0) (Armenteros et al. 2019) and the signal peptide was predicted using TargetP2.0. The transmembrane topology was predicted using a new tool DeepTMHMM (https://dtu.biolib.com/DeepTMHMM) (Hallgren et al. 2022). The secondary structures were computed using the Chou and Fasman Secondary Structure Prediction server (http://www.biogem.org/tool/chou-fasman/) (Kumar, 2013). The hydrophobicity and hydrophilicity of FIPs analysis was carried using the software BioEdit 7.2.5 with the window size set at 9 using the Kyte–Doolittle scale mean hydrophobicity profile (Hall 1999; Li et al. 2019).

#### Motif analysis

The conserved domains of FIPs of the edible mushrooms were analyzed by MEME online tool (https://meme-suite.org/meme/tools/meme) (Bailey et al. 2015), and the maximum number of motifs to identify was set to 6. The motifs were subjected the CDD-NCBI (https://www.ncbi.nlm.nih.gov/Structure/cdd/wrpsb.cgi) to know their functions.

### Alignment and sequence conservation

The graphical representation of the sequences pattern is generated using the Weblogo. The conserved patterns of amino acids with respect to their positions in the FIP family were analyzed by the sequence logo (SL). SL is a graphical representation of each amino acid position derived from the multiple sequence alignment (MSA) of the FIP family. The sequence logo was generated by using WebLogo 2.8 (https://weblogo.berkeley.edu/logo.cgi) using the MSA ClustalOmega (https://www.ebi.ac.uk/Tools/msa/clustalo/) (Crooks et al. 2004) without compositional bias.

### Gene structure analysis

The Gene Structures Display Server (GSDS) (http://gsds.cbi.pku.edu.cn/) was used to depict the exon-intron structure for available FIPs by comparing their (CDS) cDNAs and the corresponding genomic DNA sequences (Guo et al. 2007).

### Toxicity studies

The toxicity of the proteins was performed using ToxinPred2 (https://webs.iiitd.edu.in/raghava/toxinpred2/batch.html) using the default parameters of hydrid (RF + MERCI + BLAST) as a machine learning model with a threshold as 0.6.

### Phylogenetic analysis

The 19 sequences were subjected to the MUSCLE of MEGA11 for the alignment thus obtained were used for the construction of phylogenetic analysis using the neighbor-Joining method (NJ) of MEGA11 software (https://www.megasoftware.net/) and the statistical confidence was assessed with the bootstrap value set to 1000.

## Molecular docking studies

### Structure retrieval

Peptide (FIP-Glu; PDB ID: 3F3H) and protein (PDB ID: 4GUP) were retrieved from the Protein Databank (PDB) (https://www.rcsb.org/).

### Docking

The receptor molecule (MHC class I; major histocompatibility complex class I) and the peptide (FIP-Glu) were used as the ligand in the PatchDock (https://bioinfo3d.cs.tau.ac.il/PatchDock/php.php) (Schneidman-Duhovny et al. 2005) and ClusPro2.0 (https://cluspro.org/login.php) (Kozakov et al. 2013, 2017; Vajda et al. 2017; Desta et al. 2020) webservers to obtain more accurate binding energy profiles for the peptide-protein complexes. The interaction analysis was conducted using *PyMOL* (https://www.pymol.org/pymol.html).

## Results

### Physical, chemical, and structural characteristic features of FIPs

**Fig. 1:**
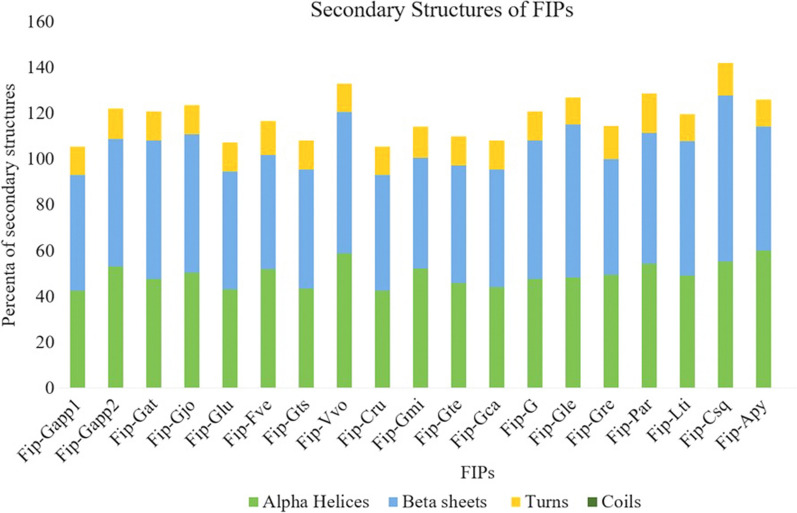
Percentage distribution of secondary structures (alpha helices, beta-sheets and coils)

Nineteen fungal immunomodulatory proteins from different edible mushrooms were identified after the search and confirmation of candidates, and they were named FIP-Gsp (G – Genus’s name and sp – species’s name; for instance, FIP of *G. lucidum* was named as FIP-Glu). As shown in Table 1, the length of the proteins ranged from 110 (FIP-Gts) – 134 (FIP-Gmi) amino acids. Furthermore, the molecular weights of these FIPs proteins ranged from 12327.66 to 15170.79 Da of FIP-Csq and FIP-Gmi respectively. Although the deduced FIPs proteins showed diversity in terms of the parameters mentioned above, 50% (11 FIPs) had (pI > 4.44) while the remaining 11 FIPs had isoelectric point (pI > 5.13) with an average of 5.85) as shown in Table 1. 3 of the 19 sequences contain signal peptides while none of the sequences contain transmembrane regions. The subcellular localization of the sequences predicted that most of the sequences (95.4%) were extracellular except one FIP (FIP-Ppl) which is cytosolic while two of the FIPs namely FIP-Gapp2 and FIP-Fve were also cytosolic and chloroplast along with the extracellular location (Table 1). The FIP motif (PF09259) was used as the query sequence to search for a similar fungal protein sequence in the HMMER program. The search was performed to identify the FIP family members. The SMART tool was then used to confirm whether the candidates contained the domain (Additional file 3: Table S1). Interestingly, it was observed that three species (namely FIP-Tve, FIP-Pco, and FIP-Tfu) ranging from 135 to 194 amino acids in length reported being FIP did not contain any FIP domain (Additional file 3: Table S2 and S3A). The FIPs were also subjected to the prediction of secondary structures. All the FIPs contain ${\upalpha }$ helices, ${\upbeta }$ 1 sheets, and turns (Fig. 1) which are similar to the other FIPs (Additional file 3: Table S3B). The sequences were subjected to instability index (II) computation and it was found that all the protein sequences are stable (< 40 II) and none of the sequences contain any transmembrane helices (signal peptides, mitochondrial or chloroplast).

**Fig. 2:**
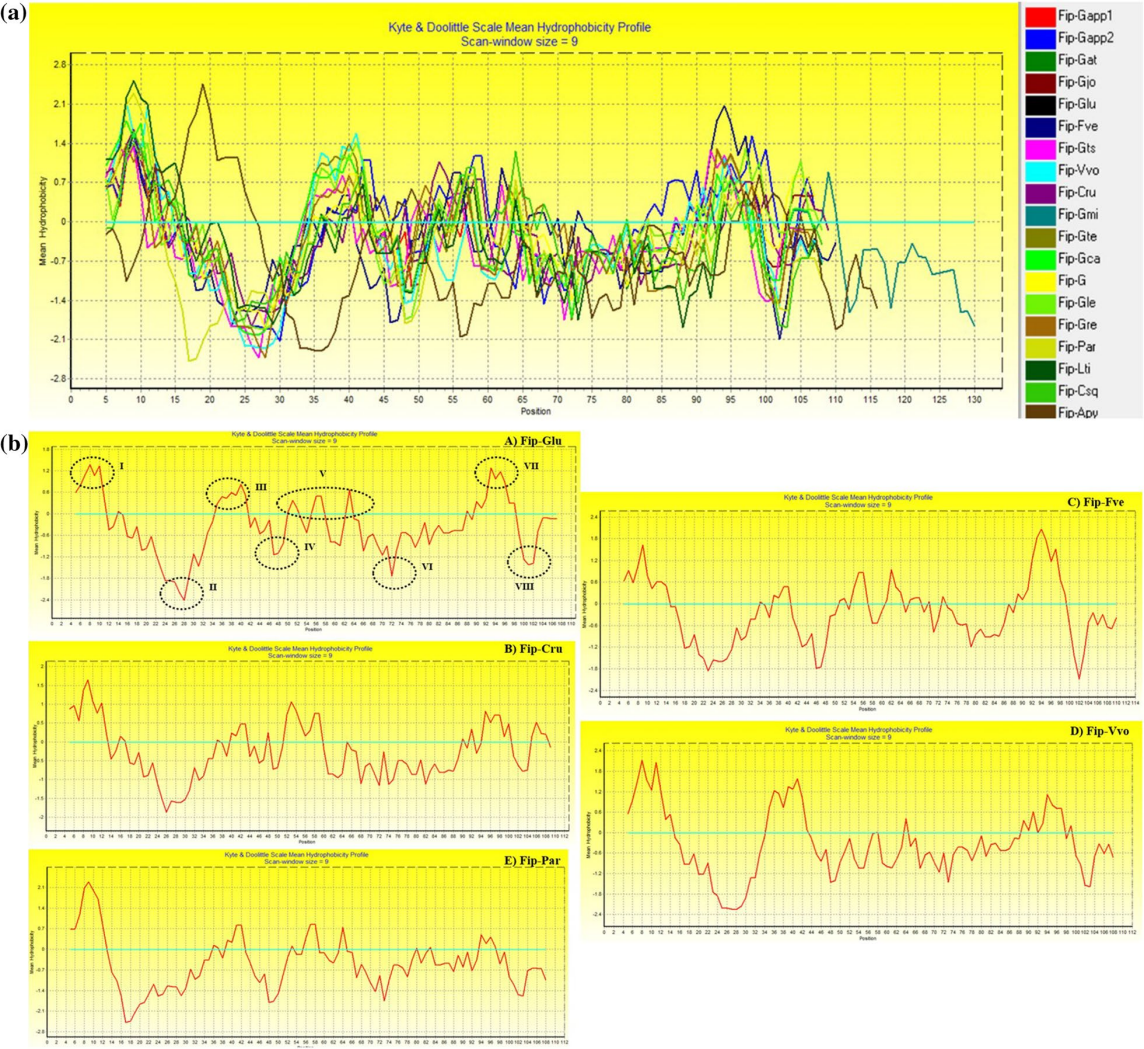
**A **Hydrophobicity profile of FIPs. The negative scores denote hydrophilicity while the positive scores depict hydrophobicity. **B** Comparison of hydrophobicity profiles of 5 FIPs namely Glu, Vvo, Fve, Par and Cru

The FIPs contain both hydrophobicity and hydrophilicity regions in their sequences (Fig. 2a). It was observed that all of the sequences have more hydrophilic in nature as compared to the hydrophobic regions. For comparison, FIPs from Glu, Cru, Par, Vvo and Fve were taken and marked regions of hydrophobicity (I, III, V, VII) and hydrophilicity (II, IV, VI, VIII). The region I found to be the highest hydrophobicity value for all the FIPs. The FIPs Glu and Cru have similar profiles except that values are less. The other three FIPs show varied profiles for the eight regions (Fig. 2b). This property will be very useful in drug delivery methods and designing novel therapeutic alternatives. Moreover, the differences in the hydrophobicity and hydrophilicity between FIPs might influence their structural stability, and also lead to differences in their functional behavior which can be depicted as recently several recombined FIPs are being developed.

### Multiple sequence alignment

**Fig. 3:**
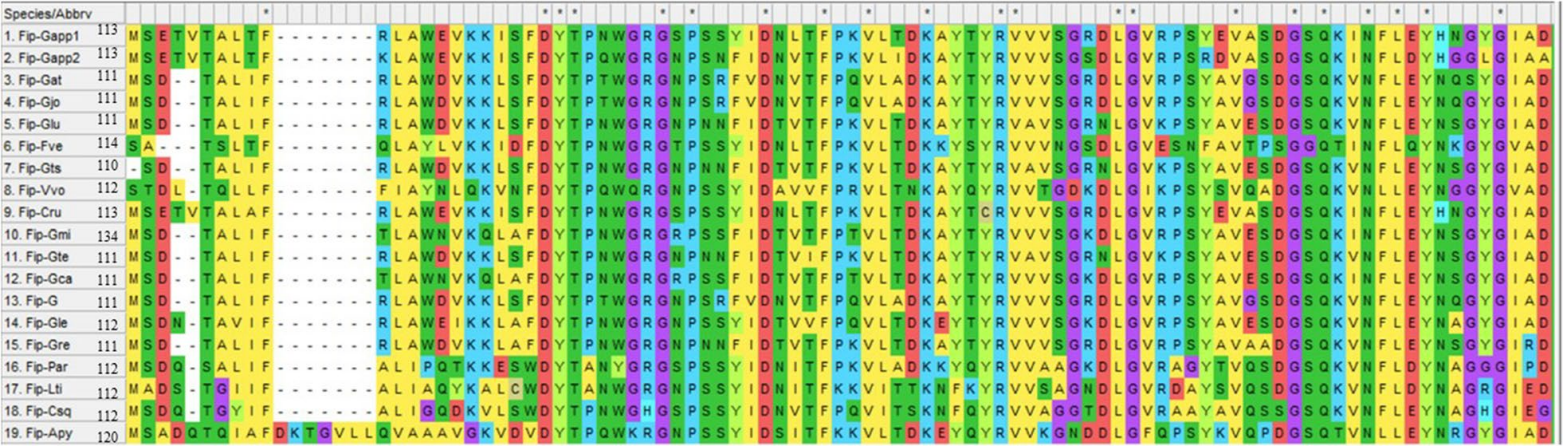
Amino acid sequence alignment of known edible fungal immunomodulatory proteins (FIPs)

**Fig. 4:**
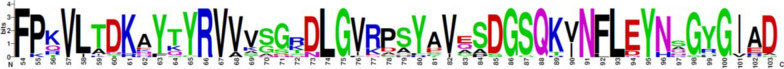
Web logo analysis of fungal immunomodulatory proteins (FIPs)

FIPs are highly conserved eukaryotic proteins and different FIPs exhibit homology. Hence, homology between different FIPs was primarily analyzed by amino acid sequence alignment using MUSCLE of MEGA11 software. The NCBI’s protein BLAST showed that the FIPs shared varied range of homology from 42.86 to 100% (Fig. 3). For instance, 42.86% between FIP-Ppl and FIP-Csq and 100% between FIP-Glu and FIP-Gts. FIP-Gat, FIP-G, FIP-Gjo, FIP-Glu, FIP-Gte shared 99.1% identity. Furthermore, FIP-Cru shared 98.23% homology with FIP-Gapp1. A weblogo was designed based on the MSA (Fig. 4).

## Motif analysis

**Fig. 5:**
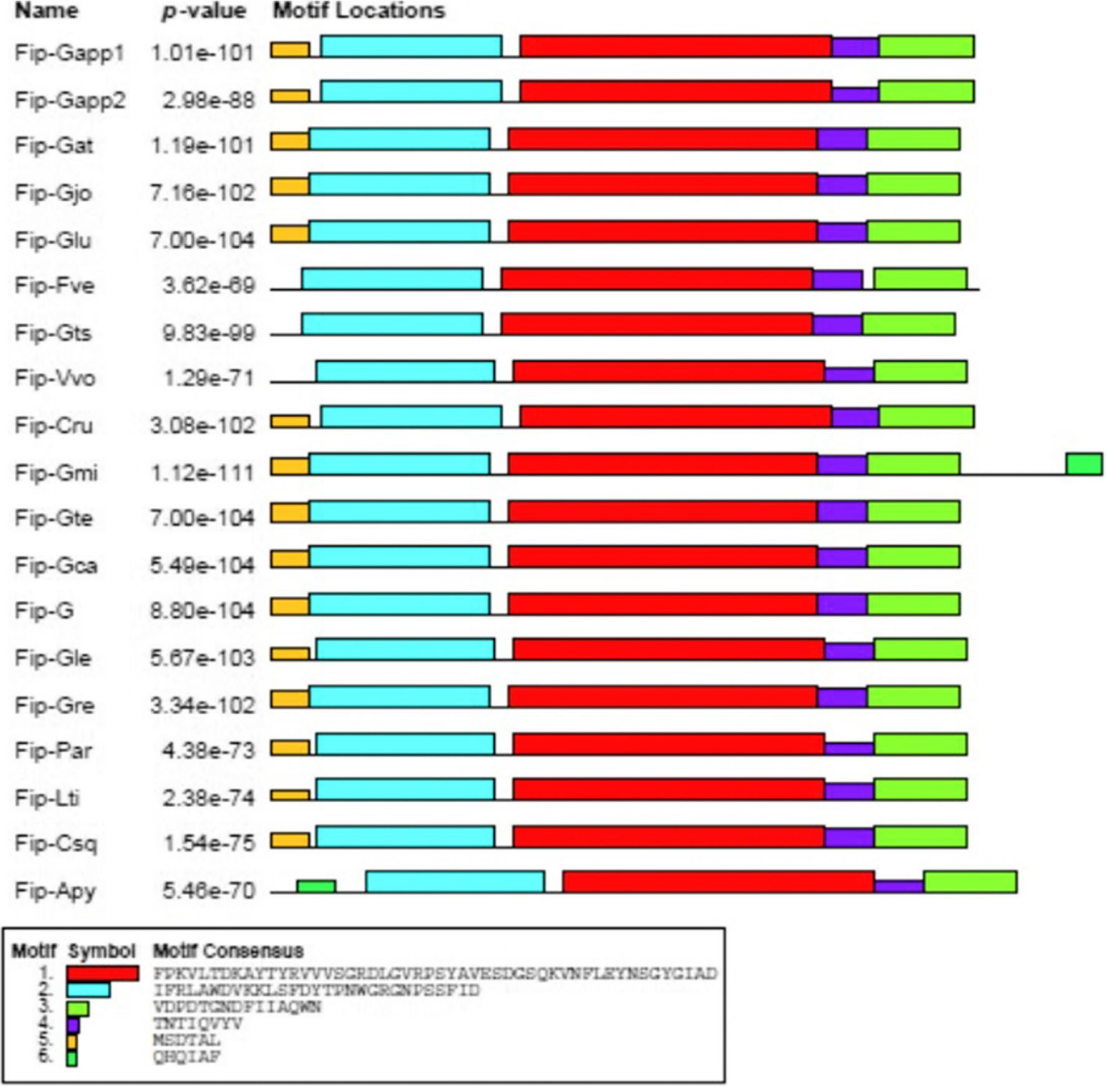
The motifs in FIP proteins of different edible mushrooms. Each motif was represented by different colored box. The sequence of each motif is listed below with the same color

The MEME (Multiple Em for Motif Elicitation) online tool was used to perform the protein motif analysis and identified the distribution of the motifs. The sequences were compared, and similar sequence motifs were calculated (Figs. 4 and 5). This combined high conservation patterns with motifs from one to six. The homology of motifs in different species means that the structural gene characteristics differ concerning exon-intron relationships. These analyses showed that the variances in the sharing of motifs in these fungal species’ proteins might have deviated from those genes’ functions during adaptive evolution. The putative functions of the motifs were analyzed using the NCBI-CDD and found that only two of the six motifs contain the FIP while the rest could be novel motifs and need to be investigated (Additional file 3: Table S4).

### Gene structure analysis

To gain further insights into the structural diversity of FIPs, the exon/intron organization in the coding sequences of the available protein sequences was studied. It is interesting to note that the intron/exon structure of FIP-Gapp1, FIP-Gapp2 and FIP-Gts are non-intronic genes while FIP-Glu contains 3ʹ and 5ʹ UTRs (Additional file 1: Fig. S1).

### Toxicity analysis

The toxicity analysis of the proteins using the ToxinPred2 (Sharma et al. 2022) showed that among the 19 sequences, only 3 are found to be toxic. The ToxinPred2 uses machine learning algorithm (Random Forest modeling) which predicted the RF score to be 0.66, 0.62, and 0.66 for FIP-Gts, FIP-Vvo, and FIP-Gmi while the Motif-EmeRging and with Classes-Identification-based and BLAST scores are 0 for all the sequences.

### Phylogenetic analysis

**Fig. 6:**
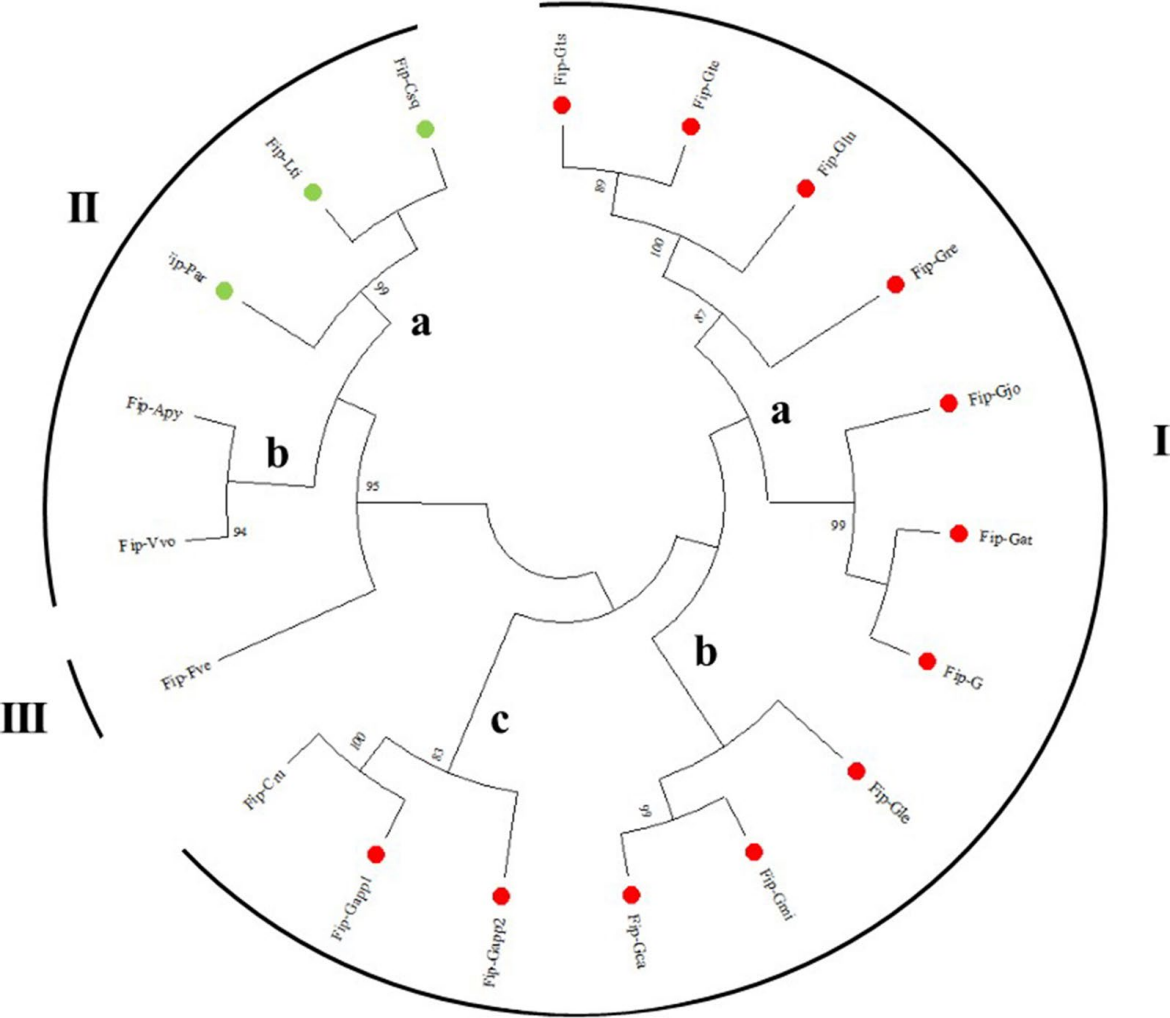
Evolutionary relationship among identified FIPs. Phylogenetic tree of FIPs constructed by MEGA11 software using the neighbour-joining (NJ) method. The red dots show members of the family Ganodermataceae and green dots depict members of the family Polyporaceae. The tree is divided into subfamilies (I, II and III) based on the homology (read text for more information)

The phylogenetic trees depict the pattern of evolutionary history that evolved from a common ancestor. It enables to ascertain speciation events by comparing their sequences’ similarities and differences. The tree of 19 FIPs was constructed, and they were divided into three subfamilies [subfamily I (a–c), II (a, b) and III; Fig. 6] based on an 80% cut-off value for the condensed tree. Subfamily I contained 13 members (12 species of *Ganoderma* and 1 species of Gomphidiaceae; *Chroogomphis rutilus*) and was the largest group, and subfamilies II contained 6 (Polyporaceae, Auriscalpiaceae and Plutaceae members), and III 1 respectively based on the homology. The topology of the tree generated using MEGA11 with the available FIP sequences revealed that the sequences of *Ganoderma* occurred in a large clustered clade (main lineage) representing ancestral similarity which is also evident belonging to the same family Ganodermataceae and simultaneously indicating the divergence from a common ancestor. This clade (I) also indicates that members of *Ganoderma* genus are relatively conservative during evolution. Among the members of the Ganodermataceae, both FIP-Gapp1 and Gapp2 occurred together (as showed by Qu et al. 2018) while However, Fip-Gapp1 and Fip-Gapp2 formed an interesting lineage with FIP-Cru and shared their similarity it, a member of the family Gomphidiaceae, which is also evident from the BLAST2Sequence (Additional file 2: Fig. S2) sharing a similarity of 98.23% with Fip-Gapp1. The Fip-Gjo shares 98.2% with Fip-Gat and 99.1% with FIP-G thereby forming a separate clade. Similarly, Fip-Gju is showing relatedness with two members of the *Ganoderma* namely FIP-Gts and FIP-Gte with 100% and 99.1% similarity respectively. Similarly, the FIPs from the family Polyporceae formed a separate clade. The FIPs from *V. volvacea* is forming a lineage with *A. pyxidatus*. The FIP from *F. velutipes* formed a unique and separate lineage, indicating a substantial phylogenetic distance between them and the FIPs from other fungi.

The tree showed interesting observations. For instance, members of the families Ganodermataceae and Gomphidiaceae from the orders Polyporales and Botetales respectively occur in the same subfamily I. However, members of the same order Polyporales but different families (Ganodermataceae and Polyporaceae) occurred in different subfamilies I and IIa. This probably would have resulted due to shuffling of the nucleotide sequences during speciation. Likewise, members of different families shared similarities. For instance, Auriscalpiaceae member species (*Artomyces pyxidatus*) (Order – Russulales) has occurred in the same subfamily IIb as *V. volvacea* from the family Plutaceae (Order – Agaricales) but yet they shared distant homology with *F. velutipes* belonging to the family Physalacriaceae (Order – Agaricales) (Fig. 6). The evolution of FIPs is unique in a way that their pattern of evolution or divergence is not limited to a particular order or family but diversified in nature. As it is also evident from previous reports that FIPs occur in edible fungi along with non-edible and medicinal fungi as well. Therefore, more studies are required to confirm the evolution of the FIPs which can also define the evolution of other FIP types.

### Docking analysis

**Fig. 7:**
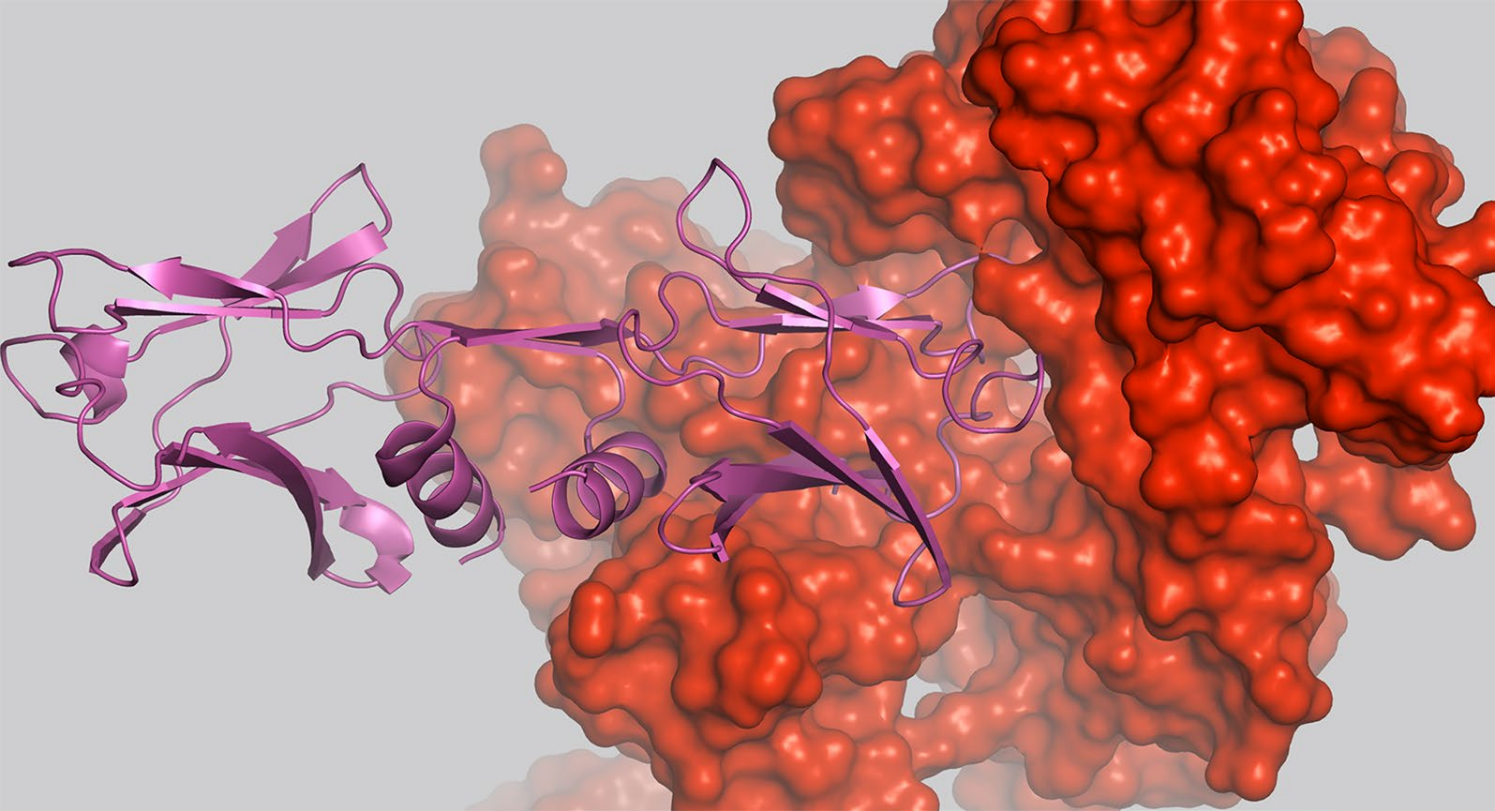
The protein-protein interaction between FIP and MHC. The FIP-Glu (LZ8) is shown in surface representation in red color and the MHC is shown in pink, cartoon form

The FIP of Glu was taken as a representative protein target and the human major histocompatibility complex class I (MHC) was taken as the receptor for the study (Fig. 7). With the application of in silico-based approaches such as molecular docking, binding affinities of various ligands for the target protein can be identified which will further aid in the development of novel drugs. The human MHC class I molecules are known to be involved in the prevention and working against tumors and/or during virus attacks. They comprise of both classical and non-classical human leukocyte antigens (HLA – A, B, C, E, G and G) (Garcia-lora et al. 2003). Similarly, FIPs are also involved in boosting immune responses and found to be associated with anti-inflammatory, anti-tumor effects and hemagglutination (Qu et al. 2018; Li et al. 2019; Chalamaiah et al. 2018). Therefore, the current study will aid in improvising the immune system using the FIPs as model protein sources in humans against the invading pathogens.

The PatchDock revealed that the score of the compound is 22248 (represents geometric complementarity score) representing molecular surface of the molecule/protein which can be used for docking (Duhovny et al. 2002) with area 3652.2 which represent the interface area of the complex. The atomic contact energies (ACE) is the energy required for transferring different atoms from water molecules to a protein and which is rapid and accurate for the estimation of solvation energy thereby can be used for further analysis (Zhang et al. 1997). Here, the ACE is 262.34 Kcal/mol. The root mean square distance (RMSD) is set to default value of 4 Å. Similar results were obtained from the ClusPro, the cluster with the lowest energy was chosen (0), the higher the value in negative, the higher is the affinity (**− **742.9) which calculated the balanced interaction energy between two proteins using Kozakov et al. (2017) (Kozakov et al. 2017). Therefore, as revealed using the toxicity study, the FIPs can be used for future drug targets and development of newer drugs and enhancing immune responses.

### Discussion and future prospects

The BLAST results revealed other fungi as well that share similarity with the FIP-Gapp1 which include *Dichomitus squalens* [Polyporaceae], a white-rot causing non-edible fungus, with 68.52% PI. Interestingly, it also shared similarity (62.93–69.16%) with several members of the genus *Fusarium* (*F. venettenii*, *F. solani*, *F. oligoseptum*, *F. kuroshium*, *F. ambrosium*, *F. solani-melongenae*, *F. keratoplasticum*, *F. duplospermum*, *F. decemcellulare* and *F. albosuccineum*) [Nectriaceae] which are known to be phytopathogenic and cause diseases in economically important crops (Rampersad, 2020; Yang et al. 2022). FIP-Gapp1 shared identity (63.39%) with other pathogenic fungi which causes black rot, *Thelonectria olida* [Nectriaceae] (Berlanas et al. 2020), *Amylostereum chailletii* [Amylostereaceae] causing white rot and shows symbiotic association with wood-boring beetles (Woodwasps; Siricidae) (Boddy 2016) and *Paramyrothecium foliicola* [Stachybotryaceae] (Sun et al. 2021). It shares similarity (56.64%) with non-edible yet medicinally important fungus, *Lenzites betulina* [Polyporaceae] (Sarma et al. 2010), *Trametes sanguinea* [Polyporaceae] (Kakoti et al. 2022), *Botryobasidium botryosum* [Botryobasidiaceae], *Stachybotrys chartarum* [Stachybotryaceae] (Li et al. 2016; Hyde et al. 2018) and *Stachybotrys chlorohalonata* [Stachybotryaceae] (Semeiks et al. 2014). It also showed identity with *Polyporus brumalis* [Polyporaceae] and *Postia placenta* [Postiaceae], inedible fungi (Phillips, 2010; Li et al. 2015). This shows that FIPs are also present in a wide variety of fungi including non-edible and medicinally important species and therefore, need to studied and investigated further (See Box 1 for Outstanding Questions).

The bioactivities of FIPs either derived from edible or medicinal mushrooms, their roles have been qualitatively determined to inhibit cancer (Li et al. 2017; Lin et al. 2021; Li et al. 2022), hemagglutinate blood cells, and selectively enhance the expression of mRNA of cytokines (TNF-a, IL-1, IL-6, and IL-12) in spleen cells and in human peripheral blood lymphocytes [other roles have been reviewed by Lin et al. (2022)]. Therefore, FIPs are good candidates for developing novel drugs or new types of functional food supplements for treating and preventing different kinds of diseases including cancer, etc. The knowledge and development of commercial development of FIPs is however scant. The docking will be useful in order to enhance or boost immune system to work against the invading pathogenic microbes. Due to the limitations and difficulty in directly obtaining them from mushrooms which require long protocols for extractions and are also cost ineffective with low yields. Genetic engineering could provide an efficient method for the mass production of these bioactive proteins.

The study is preliminary and is related to the study of nutritional and health benefits of the FIPs obtained from mushrooms. It can be conducted on a large scale to get more accurate results and to validate them statistically and in in vivo conditions. Moreover, there is a lack of awareness about the nutritional benefits of mushrooms among people, and therefore, needs to be emphasized. The fungal immunomodulatory proteins are an important source that has many properties which can be used for treating ailments and diseases, therefore, this area needs to be explored.

Outstanding questions (Box 1)

1. Complete annotation of the genomes is not available which makes it difficult to understand and investigate about the FIPs in more depth.
2. Sequence information is not yet completely accessible for the FIPs.
3. FIPs are present in a diverse and wide range of fungi including non-edible and medicinal. The exact structural and functional features are not yet understood clearly. What is significance of FIPs in non-edible fungi? There is an urgent need for the elucidation of mechanism and roles of FIPs from the medicinal fungi.
4. As the FIPs yet to be discovered from other fungi too and there are missing links about their structural and functional properties, for instance, the other types of FIPs (Cerato-type FIPs, PCP-like FIPs, TFP-like FIPs, and unclassifiedFIPs) and their relation with Fve-type FIPs is required for unraveling precise mechanism of their biological activities. Furthermore, only three structures are available for the Fve-type FIPs which also hinders further studies in this direction.
5. Evolutionary studies are still in its infancy stage due to several missing gaps in understanding FIPs.
6. This study also shows that some FIPs are classified as FIP did not contain the Pfam identify, how they have been classified as FIPs?

## Supplementary Information

**Additional file 1: Fig S1.** Exon-intron structure of some selected FIPs.

**Additional file 2: Fig S2.** Percent identity matrix of the fungalimmunomodulatory proteins (FIPs).

**Additional file 3: Table S1.** SMART analysis of the FIPs. **Table S2.** Sequence properties ofedible mushrooms containing other type of FIP. **Table S3.** A) SMART analysis ofthe other FIPs types and B) Secondary structure analysis of other FIPs types. **Table S4.** FIPs motifs and theirputative functions.

## Data Availability

The data is provided in the manuscript.
