# Supplementary figures and images for: In Silico functional and phylogenetic analyses of fungal immunomodulatory proteins of some edible mushrooms
Source: AMB Express. 2022 Dec 26;12:159. doi: 10.1186/s13568-022-01503-w (PMC9791630; doi:10.1186/s13568-022-01503-w)

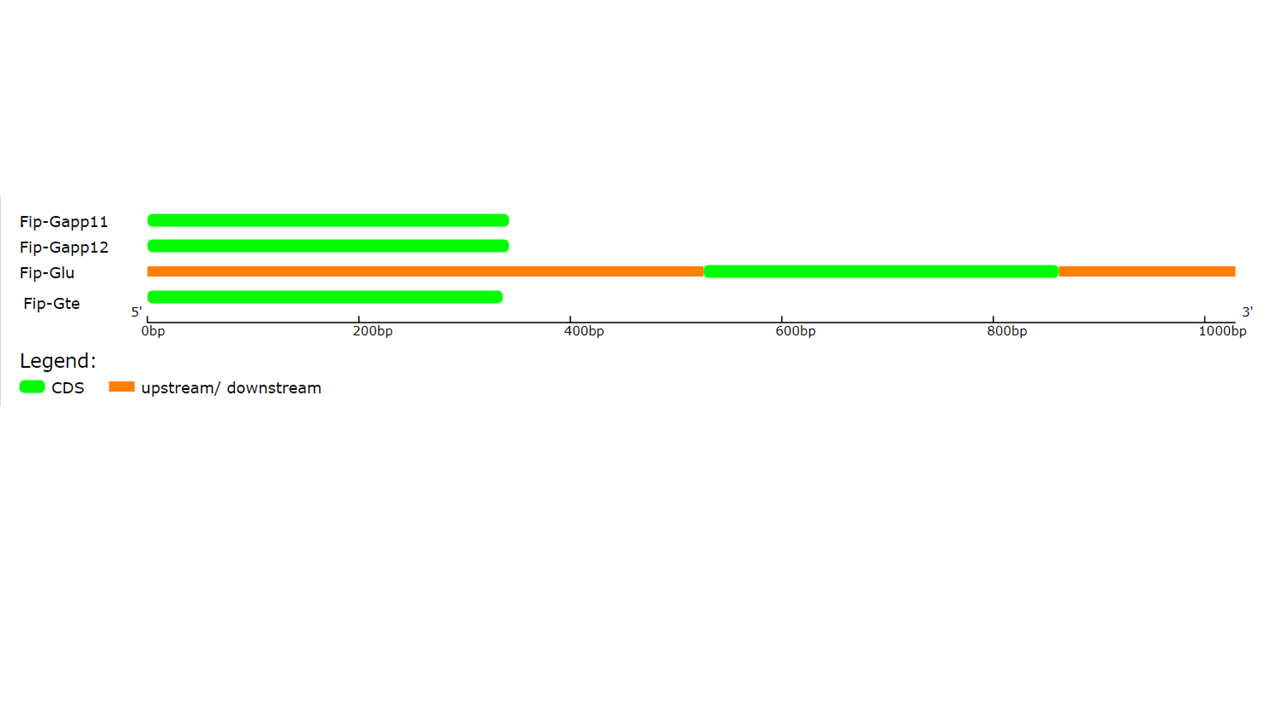

Supplement: Supplementary file 1 — Additional file 1: Fig S1. Exon-intron structure of some selected FIPs. [file 13568_2022_1503_MOESM1_ESM.png]

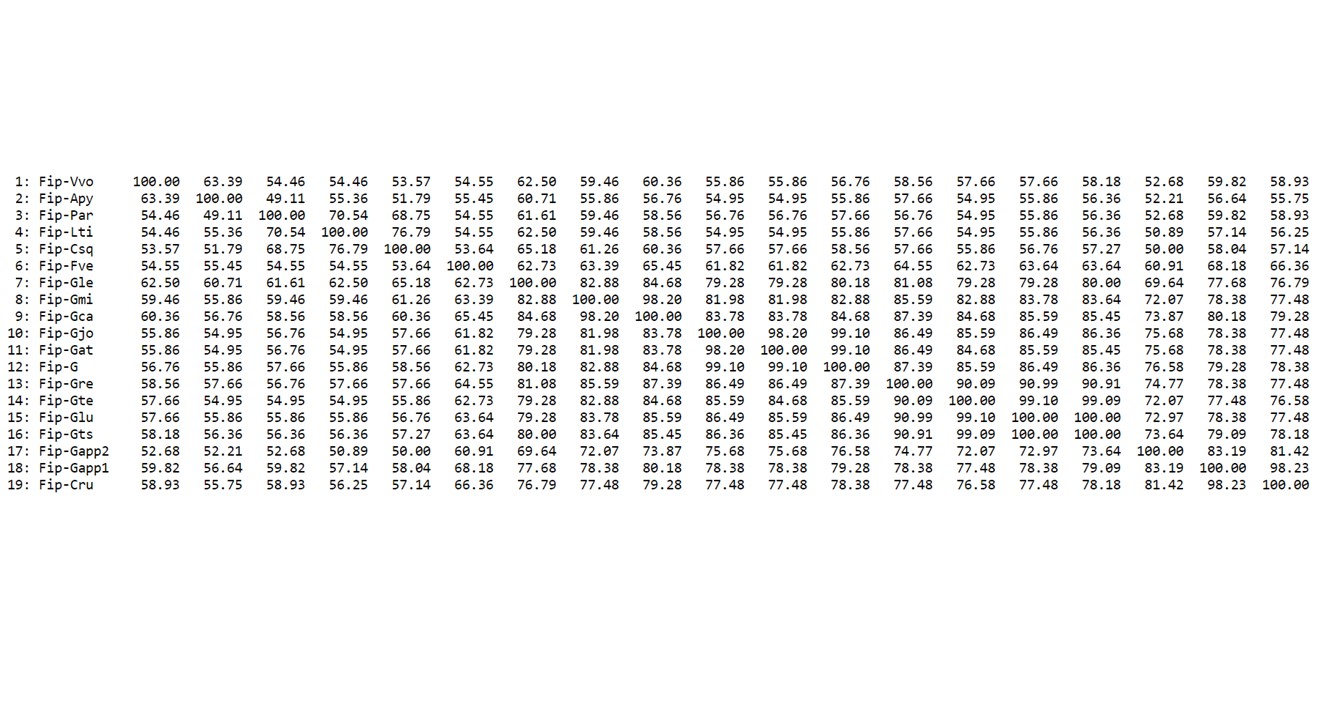

Supplement: Supplementary file 2 — Additional file 2: Fig S2. Percent identity matrix of the fungalimmunomodulatory proteins (FIPs). [file 13568_2022_1503_MOESM2_ESM.jpg]
